# Method to determine the nadir PSA following partial gland ablation

**DOI:** 10.1002/bco2.496

**Published:** 2025-02-14

**Authors:** Nelson N. Stone, Vassilios Skouteris, Rendi Shu, Richard G. Stock, Ben GL Vanneste

**Affiliations:** ^1^ Department of Urology The Icahn School of Medicine at Mount Sinai New York New York USA; ^2^ Department of Radiation Oncology The Icahn School of Medicine at Mount Sinai New York New York USA; ^3^ Brachytherapy Center Hygeia Hospital Athens Greece; ^4^ Department of Radiation Oncology (Maastro), GROW School for Oncology and Reproduction Maastricht University Medical Centre+ Maastricht The Netherlands; ^5^ Department of Human Structure and Repair; Department of Radiation Oncology Ghent University Hospital Ghent Belgium

**Keywords:** biochemical failure, biopsy, focal therapy, nadir PSA, prostate cancer, radiation dose

## Abstract

**Objectives:**

The objective of this study is to propose a novel method of determining the nadir PSA (nPSA) for men with prostate cancer treated by partial gland ablation (PGA).

**Materials and Methods:**

Two cohorts of men were analyzed to develop a formula for the nPSA in men undergoing PGA. First, 123 men with a suspicion of prostate cancer underwent transperineal mapping biopsy (TPMB) and found to have benign pathology. Their prostate‐specific antigen (PSA) was compared to the prostate volume using curve estimation regression analysis. Second, the contribution of PSA from an ablated region was determined by using a surrogate of 545 men who had whole‐gland brachytherapy followed by prostate biopsy. Biopsy results were compared to radiation dose (calculated as the biological equivalent dose) levels in men who were free from biochemical failure. The nPSA was then calculated by using the PSA density (PSAD) for the untreated volume plus the PSA from the post‐brachytherapy patients.

**Results:**

The PSAD with the highest *R* ^2^ (0.80, *p* < 0.001) for the 123 men who had TPMB and a negative biopsy was 0.12 ng/mL^2^. In the brachytherapy patients, five 20 Gy dose groups were analyzed from ≤140 to ≥220 Gy, which demonstrated a progressive decrease in the positive biopsy rate to 1.5% at the highest dose (*p* = 0.036). PSA was <0.2 ng/mL in 98.2% of these men. If brachytherapy was used for PGA and a dose of ≥ 220 Gy was delivered to the ablation zone, the nPSA could be calculated from the remaining untreated volume as: the [(pretreatment PV)–treated volume] ×0.12 ng/mL^2^.

**Conclusion:**

A method for determining the nPSA following PGA using brachytherapy was developed. The formula relies on complete ablation of the treated volume, which resulted in no PSA contribution from that component. Other forms of ablative energy should yield similar results. Further clinical validation of this concept is warranted.

## 1 INTRODUCTION

Prostate cancer is one of the most common cancers in men worldwide, with an estimated 1 500 000 cases and 375 000 deaths in 2020.^1^ Different strategies for the management of localized prostate cancer include radical prostatectomy, brachytherapy or external beam radiotherapy; however, each has their own side effects.^2^, ^3^ Active surveillance (AS) has also become an increasingly popular approach for low‐ and low‐intermediate prostate cancer with the desire to avoid side effects. However, upwards of 50% of men placed on AS eventually receive treatment, either because of subsequent upgrading or patient choice.^4^, ^5^ Focal or partial gland treatment is another option being explored, but unlike prostate gland removal or radiation therapy where success is measured by a PSA < 0.2 ng/mL for the former and an increase to >2 ng/mL above a nadir (Phoenix failure) for the latter, no standard definition for biochemical failure exists for partial gland ablation (PGA). In a systematic review of focal therapy, Valerio et al. reported that of a total of 2350 treated men in 30 studies most investigators used post‐treatment biopsies to determine success, while a minority defined biochemical control using Phoenix criteria (nadir plus 2 ng/mL), ASTRO failure definition (three rises above the nadir), Stuttgart (nadir plus 1.2 ng/mL) and Phoenix in combination with PSA velocity >0.75 ng/mL per year.^6^ The lack of a consensus on what the nadir PSA (nPSA) should be after PGA is one of the factors hindering further adoption of a focal therapy PGA strategy.

Prostate‐specific antigen density (PSAD), which is the ratio of PSA to prostate volume (PV), is recognized as a prognostic marker for prostate cancer.^6^ Jue et al. found that PSA density performed better than PSA for detecting prostate cancer in men who had a previous negative biopsy (AUC: 0.69 vs. 0.56, *p* < 0.001) versus those who did not (AUC: 0.72 vs. 0.67, *p* < 0.001).^7^ PSAD has also been shown to have predictive value for assessing prostate volume, with areas under the curve ranging from 0.76 to 0.78 for various prostate volume cut‐off points, but it has not entered clinical practice other than to help identify which patients to biopsy with clinical suspicion of prostate cancer or equivocal mpMRI findings.^8^

To determine the nPSA following PGA, the amount of PSA contributed by the untreated portion of the gland needs to be combined with the amount of PSA released by the treated volume. However, there is little information available on how much PSA is released from the treated zone. Various studies that report infield recurrences (biopsy or MRI) will also confound the ability to know the actual PSA in this region.

Prostate brachytherapy provides a good model to better understand these relationships. Previous studies demonstrate the relationship between negative post‐treatment (brachytherapy biopsies), undetectable PSA levels and different biologically effective dose (BED) can serve as a surrogate for determining if any PSA remains after complete ablation.^9^

We investigated a method of calculating the nPSA after PGA by first determining the PSAD in men who had extensive transperineal prostate mapping biopsy where no prostate cancer was detected, the BED required to approximate 100% local control, and the amount of serum present PSA after this control was achieved. These factors contributed to the development of an equation that generated the nPSA after a successful focal treatment or PGA. We also describe a clinical case where this formula was applied.

## 2 MATERIALS AND METHODS

### 2.1 PSA density determination in men without prostate cancer

A cohort of 243 men with clinical suspicion of prostate cancer underwent transperineal mapping biopsies (TPMB) between 2008 and 2021. mpMRI was only recently offered and 19/23 had PI‐RADS ≥3 lesions. The mean age, PSA and PSAD were 67.1 years (95th percentile 53–81), 8.5 ng/mL (2.9–16.2) and 0.20 (0.06–0.46). TPMB was performed using the BK ProFocus with model numbers 8558/8568 transrectal biplanar probe (BK Medical, Peabody, MA, USA) under general anesthesia using an 18‐gauge disposable biopsy device (BD, Max‐Core, Franklin Lakes, NY, USA). The patient was set up in lithotomy position where the biopsies were taken through a template (CIVCO Medical Solutions, 102 1st St S, Kalona, IA 52247, USA) in 5 mm intervals as previously described.^10^ If the length of the prostate was greater than 2 cm inline, biopsies were also taken. For men with PVs greater than 50 cc, extended lithotomy (over stretching of the legs) was used to access the anterior lateral of the gland.^11^ A proprietary program was used to create intraoperative 2D and 3D prostate images from the ultrasound, to record the location of the biopsy sites and to determine prostate volume.^10^ Each specimen was analyzed for the presence, length and location of prostate cancer. Men with evidence of prostate cancer, high‐grade prostatic intraepithelial neoplasia or atypia, use of hormone therapy or 5‐alpha reductase (AR) inhibitors were excluded leaving 123 men for analysis. The PSA before biopsy was compared to the PV (determined at the time of mapping) for each patient to generate the PSADs.

### 2.2 Determination of PSA levels in the treated volume

The contribution of PSA from the treated prostate volume was determined from a surrogate of 545 men who had whole‐gland (WG) prostate brachytherapy and biopsy a mean of 6.7 years (range 2–17) after all therapy. The patients were treated with either implant monotherapy (I^−125^ or Pd^−103^), implant with short course androgen deprivation (AD, median 6 months of luteinizing hormone‐releasing hormone analogues [LHRHa] + antiandrogen) or combination brachytherapy plus external beam radiation therapy (EBRT): planning dose for I^−125^ 110 plus 45 Gy EBRT plus a median 9 months of AD (high risk patients).^12^ Radiation doses were converted to the BED using the post‐implant D90 and any external beam boost with an α/β of 2.^13^ BED levels were analyzed to determine the dose necessary for a negative biopsy and an undetectable PSA (<0.2 ng/mL). The BED for patients treated with combination therapy was calculated as the sum of the BEDs from the implant and EBRT.^13^

### 2.3 Focal brachytherapy procedure

A 73‐year‐old with a pretreatment PSA of 7.0 ng/mL (T1c) underwent transperineal mapping prostate biopsy. Of the 46 samples, three were positive for Gleason grade group two involving both posterior lobes. The patient was offered surveillance and definitive therapy and opted for PGA with I^−125^ brachytherapy. He underwent focal therapy on 21 September 2021 with a prescription dose of 200 Gy to the clinical treatment volume (CTV‐both posterior lobes), which were defined by negative biopsies outside this region. Androgen deprivation was not administered.

### 2.4 Statistical analysis

PSA (dependent variable) was compared to the PV (independent variable) by curve estimation using regression analysis for the men with benign disease as determined by the TPMB. Three models were run: linear, logistic and logarithmic. Two sets of data were analyzed, for all 123 men and a group that removed outliers that consisted of the 75 percentile of the PSAD values of the entire group (*n* = 62). The median PSAD for both groups was used for the final formula.

To determine the contribution of residual PSA in the treated volume and the dose required to achieve complete local control three BED categories, <150, 150–200 and >200 Gy and five 20 Gy dose groups from <140 to ≥220 Gy and were analyzed and compared to the biopsy results and PSA levels using ANOVA and chi‐square tests. The PSA contribution from the untreated PV was combined with the PSA from the treated volume to calculate the nPSA following PGA. Statistical analyses were performed using SPSS v.26.

## 3 RESULTS

**FIGURE 1:**
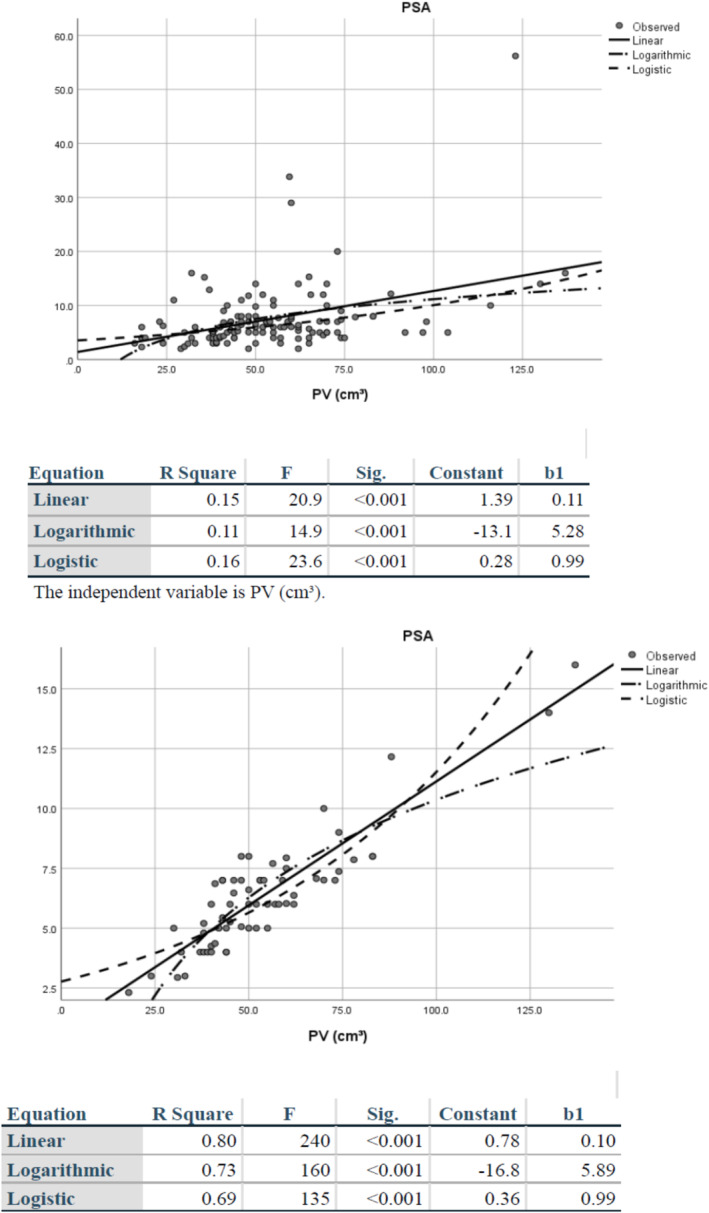
(a) Curve estimation regression for entire cohort of 123 men with benign disease. (b) Curve estimation regression for the restricted cohort of 62 men.

One hundred twenty‐three (55.1%) of 243 men with clinical suspicion of prostate cancer who underwent TPMB were found to have benign pathology only. The median PSA and PSAD were 6.0 and 0.12 ng/mL for these men and the 62 men within the 75th percentile of PSAD values (table 1). The men in the entire cohort had higher PSA and PSAD but similar PV (54.7 vs. 53.6 cc) and number of biopsies (50 vs. 49) as the restricted cohort. The curve estimation for the entire cohort had a *R* ^2^ of 0.15 (*p* < 0.001), while it was 0.80 (*p* < 0.001) for the restricted cohort (Figure 1a,b). The median PSADs for both datasets were 0.12 ng/mL. There were 99 men who had a PSAD ≤0.17 ng/mL^2^ who were followed a median of 5.1 years (95th percentile 1–10.2). The median change in PSA was −0.5 ng/mL (−5.5 to 3.5), and no patients were subsequently diagnosed with prostate cancer.

**TABLE 1:** 123/62 men in the entire/75th percentile cohorts for prostate‐specific antigen (PSA) density.

	Age	PSA	PSAD	PV (cm^3^)	Total cores
**Mean**	64/65.1	7.6/6.3	0.146/0.12	54.6/53.6	50.3/49.2
**Median**	65/65.5	6/6	0.117/0.12	52/50	50/51
**Std. deviation**	8.1/7.2	6.4/2.4	0.098/0.02	21.9/20.6	12.7/10.7
**Minimum**	47/49	2/2.3	0.032/0.09	16/18	24/26
**Maximum**	85/80	56.2/16.0	0.569/0.17	137/137	86/74

The 545 men who had post‐brachytherapy biopsy were followed a mean of 11.3 years (range 5–24). Four hundred ninety‐seven (91.2%) had negative biopsy of which 397 (79.9%) were without Phoenix failure and 363 (73%) without AUA failure (PSA ≤ 0.2 ng/mL). The last mean PSA for the three BED groups in the men without biochemical failure according Phoenix definition were 0.11, 0.125 and 0.045 ng/mL (*p* = 0.014, Table 2). The percent of men with a PSA <0.2 was 85.9%, 90.5% and 97.3% for the three BED groups (*p* = 0.003).

**TABLE 2:** Last prostate‐specific antigen (PSA) for biologically effective dose (BED) groups <150, 150–200 and >200 Gy^2^ in men with negative biopsy and no Phoenix failure. 384/397 (96.7%) had BED calculations. Post‐treatment follow‐up was a mean of 11.3 years.

BED (Gy)	N	Mean PSA (ng/mL)	Std. deviation	Std. error	95% Confidence interval for mean		Minimum	Maximum
					Lower bound	Upper bound		
**≤150**	**56**	**0.11**	**0.19**	**0.03**	**0.06**	**0.16**	**0.0**	**0.8**
**150–200**	**179**	**0.12**	**0.33**	**0.03**	**0.08**	**0.17**	**0.0**	**1.9**
**>200**	**149**	**0.05**	**0.1**	**0.01**	**0.03**	**0.06**	**0.0**	**0.8**
**Total**	**384**	**0.09**	**0.25**	**0.013**	**0.07**	**0.12**	**0.0**	**1.9**

**FIGURE 2:**
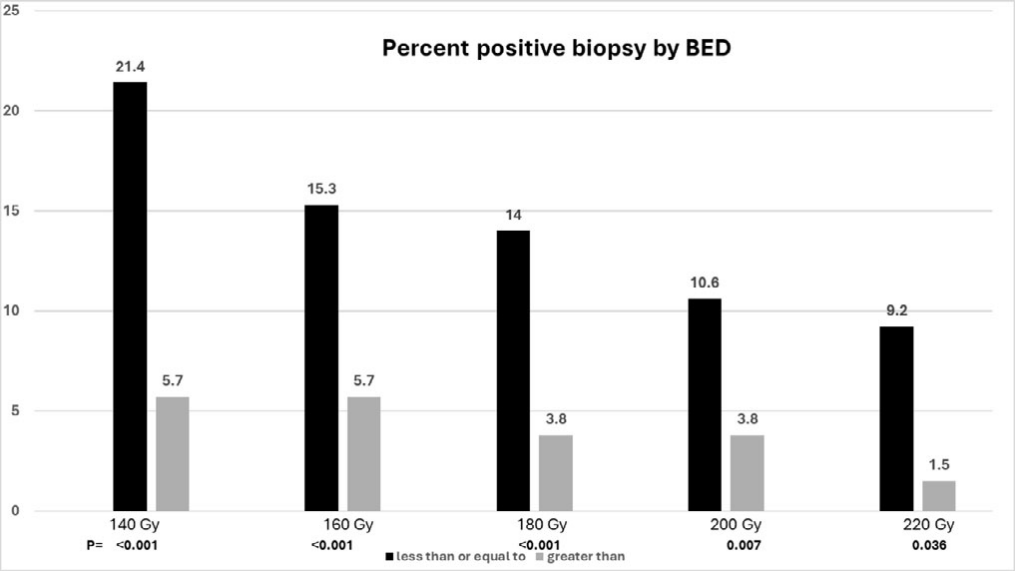
Percent positive post‐brachytherapy biopsy by dose group, *n* = 524. Each dose group is dichotomized to ≤ versus >, for example, a dose equal to or greater than 220 Gy versus all those who received a lower dose. BED = biologically effective dose.

For the 481 men with a negative biopsy the mean BED was 181.7 versus 147.4 Gy for those with a positive biopsy (*p* < 0.001). The five 20 Gy dose groups from 140 to 220 Gy demonstrated a progressive decrease in the positive biopsy rate to 1.5% once the highest BED was delivered (Figure 2). Men who had a BED ≥220 Gy had a PSA <0.2 ng/mL in 98.2% versus 91.1% for dose less than 220 Gy (*p* = 0.036).

A delivered BED of at least 220 Gy resulted in a negative biopsy rate of 98.5% and a PSA ≤0.2 ng/mL. If a partial implant to the clinical treatment volume received 220 Gy, then no or a negligible amount of PSA is produced, whereas the untreated volume should continue to produce the same PSA as a noncancerous prostate. The PSA contribution of the remaining benign prostate (untreated) could then be calculated as follows:

$$\text{nadir}\;\mathrm{PSA}=\left[\text{pretreatment}\;\mathrm{PV}\;\left(\mathrm{cc}\right)–\mathrm{CTV}\;\left(\mathrm{cc}\right)\right]\times 0.12\;\mathrm{ng}/\mathrm{mL}.$$

### 3.1 Focal brachytherapy results example

**FIGURE 3:**
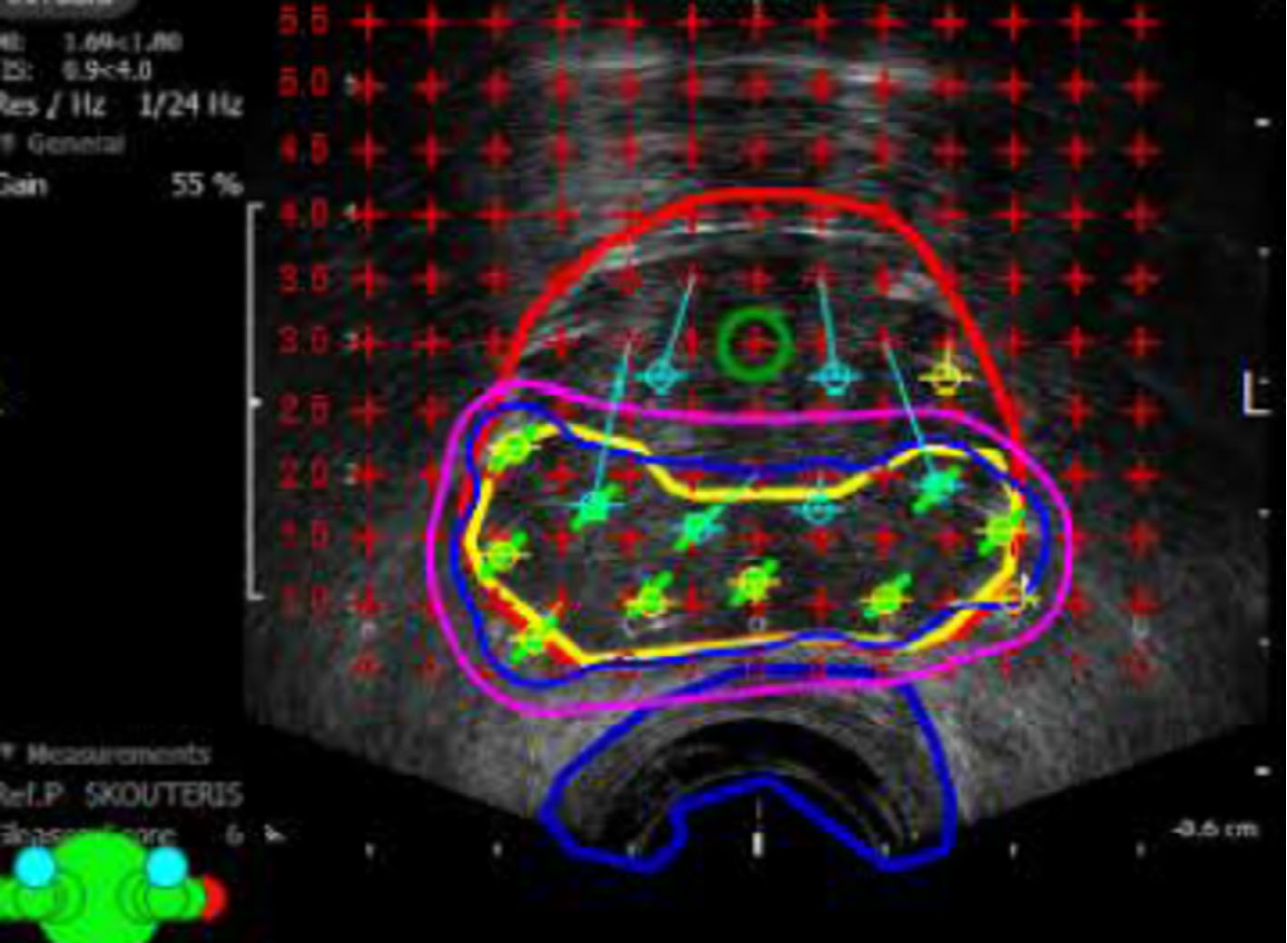
Focal brachytherapy treatment to posterior of the prostate (red contour). The yellow represents the clinical treatment volume (CTV) defined by negative biopsies outside of the CTV. The dark blue and pink lines represent the 200 and 160 Gy isodose clouds to the CTV. The biologically effective dose (BED) to the CTV was 214 Gy^2^. The green circle represents the urethra, and the blue line represents the rectum.

The patient received a BED 220.3 Gy to the clinical treatment volume, which measured 18.8 cc (Figure 3), The pretreatment PV was 40 cc, yielding an untreated volume of 40–18.8 cc = 21.2 cc. The predicted nPSA was 21.2 × 0.12 = 2.54 ng/mL. The patient's PSA decreased to 1.7 by 3 months, 1.3 at 12 months and 0.7 ng/mL by 3 years post‐implant. The pretreatment International Prostate Symptom Score (IPSS) of 13 increased to 20 in 3 months, returned to baseline at 1 year and had remained at 12 since. No proctitis was experienced.

## 4 DISCUSSION

Successful prostatectomy is expected to result in a PSA <0.2 ng/mL, while radiation therapy failure is determined by a 2 ng/mL PSA rise above a nadir (Phoenix definition). No PSA benchmark has been identified when only a part of the prostate is treated. Clinicians often resort to invasive and expensive investigations to reassure themselves that the intervention whether it be brachytherapy, cryoablation, high‐intensity focused ultrasound (HIFU) or other newer forms of ablative therapy were successful.^14^, ^15^ This study proposes a method to determine the nadir PSA after PGA that could eliminate the need for invasive testing and evaluation until the PSA increases above this level.

The elements that were investigated to create the formula relied on the contribution of PSA from the untreated noncancerous PV and from the treated PV (the CTV). The PSAD from the untreated volume was determined to be 0.12 ng/mL^2^, which was the median value from the entire and restricted cohorts, the latter having a higher *R* ^2^ of 0.8. This PSAD is similar to the report of Nath et al. who found a PSAD of 0.11 ng/mL^2^ in 52 men with BPH after TRUS biopsy.^16^ A PSAD of 0.11 ng/mL^2^ was also found in BPH patients by Tijani et al. in a similar investigation.^12^ Unlike the present study, which used an extensive TPMB to fully interrogate the pathology of the prostate, the intent of those two investigations was to differentiate the PSAD in men with benign disease from those with prostate cancer. Regardless, both studies reported PSADs of 0.11 ng/mL^2^, which compare favorably to the PSAD of 0.12 ng/mL^2^ for the men reported in the current investigation.

The contribution of PSA from the CTV was <0.2 ng/mL in 98.2% of the men without Phoenix failure if the entire gland received a BED of 220 Gy or greater. This dose resulted in nearly total ablation of both the benign prostate epithelial cells and the prostate cancer glands. The lack of PSA production in the treated area and the morphologic changes associated with higher radiation therapy have been reported by Unger et al.^17^ The conclusion from the current and previous studies is that the completely ablated tissue of the clinical treatment volume with BED >220 Gy should not produce PSA, and therefore, the only PSA produced would come from the untreated portion of the gland.

In addition to confirming the loss of PSA production from the CTV, a dose of 220 Gy also resulted in a local control rate of 98.5%. One of the concerns about offering PGA is the risk of infield and out‐of‐field recurrences. Lopez et al. reported biopsy infield recurrence in five (15%) and out‐of‐field in 11 (33.3%).^18^ Tourinho‐Barbosa et al. studied 309 patients who had focal therapy with HIFU or cryotherapy.^19^ Infield recurrences  was found in 101 patients (33%), including 66 of 219 (30%) with pre‐focal therapy biopsy findings of GS 6 and 35 of 90 (39%) with GS 7 disease. Out‐of‐field recurrences was noted in 55 patients (19%). PSA values were not used to define failure since the authors believed no data existed on how to use PSA after focal therapy. The techniques by the authors were HIFU and cryotherapy. To improve targeting, Aker et al. performed MRI‐guided cryotherapy PGA on 143 men and of 71 who had 18‐month biopsy; 35% still had clinically significant prostate cancer (csPCa).^20^ Based on the above, most centers will continue to rely on post‐treatment biopsy or MRI, a situation that requires patients to have aggressive follow‐up.

While focal therapy for prostate cancer is gaining interest most investigations have not utilized brachytherapy but rather mostly cryoablation, laser (vascular‐targeted photodynamic therapy), HIFU and irreversible electroporlation (IRE).^21^, ^22^ Ta et al. reported on 39 men with low‐ to intermediate‐risk prostate cancer treated with focal brachytherapy between 2010 and 2015. The dose prescription was 145 Gy, and failure was defined as the presence of any residual prostate cancer in the treated area. The mean follow‐up time was 65 months (range, 43–104 months). After 24 months, 34 patients underwent control biopsies. The biopsies were negative in 27 cases (79%) and positive in seven cases (21%), all outside the volume treated.^14^ Langley investigated the feasibility of hemi‐gland (HG) brachytherapy in 30 patients and compared quality of life parameters to 362 who received WG treatment.^23^ Follow‐up for HG and WG cases was 72 versus 84 (24–144) months, respectively. The IPSS, international index of erectile function (IIEF) and bowel QoL returned to baseline after HG treatment. Nadir PSA was 0.6 ng/mL in HG compared to 0.2 ng/mL for the WG patients (*p* < 0.001). Treatment failure occurred in 2 (6.7%) HG versus 20 (5.5%) respectively.

Saito et al. recruited 24 intermediate‐risk with unilateral prostate cancer for a prospective trial of HG brachytherapy with iodine^−125^ seed implantation to prescribed dose of 160 Gy.^24^ The D90 to the clinical treatment volume averaged 150.2 Gy (BED 158 Gy), which comprised 44.8% of the entire prostate volume. Mean nadir PSA 1–2 years after HG ablation was 2 ng/mL. The mean pretreatment PV was 26.8 cc. In these 24 men, the average untreated PV would have been 28.6 × 0.448 = 12.8 cc. Using the formula for nPSA from the current study, the expected PSA should have been 12.8 cc × 0.12 ng/cc = 1.54 ng/mL. The difference between the nadir PSA of 2 ng/mL reported by Saito, and the predicted one of 1.54 ng/mL may reflect the lower dose prescribed to the treated volume allowing the undertreated CTV to continue producing PSA.

Mohamad et al. recently reviewed 10 studies, which included 315 patients of which 236 (75%) had LDR and 25% HDR brachytherapy.^25^ Most studies used the Phoenix definition for PSA failure. Of the patients who had biopsy 14–46.2% had infield recurrences, most of which were residual carcinoma with radiation effect. As the biopsies were performed 12–18 months after treatment, it is difficult to know whether these indeterminate findings would revert to negative with longer follow‐up. Stone et al. reported on repeat biopsies following brachytherapy and found those that were initially positive (cancer with radiation effect) were more likely to revert to negative if the patients received a higher BED.^26^ If prostate biopsy is used following PGA to determine treatment success, it is best to wait up to 3 years after treatment before assessing the CTV.

There are several limitations to this investigation. The proposed formula should be validated in a clinical trial. The risk of Out‐of‐field recurrences also needs to be addressed. This is not an insignificant issue as these recurrences are not uncommon and will continue to make focal therapy or PGA an unattractive option compared to WG therapy if both clinician and patient have ongoing concerns about disease persistence in the untreated area. A biopsy strategy that can confirm the absence of disease in the non‐treated portion of the gland and the locations to target therapy has been published and its adoption, or something similar, could lead to a greater use of PGA.^26^, ^27^ In a study comparing mpMRI (PI‐RADS 3–5) to TPMB, the MRI had an accuracy of 53.8% compared to the mapping biopsy in the detecting the locations of csPCa when the gland was divided into four sectors (quadrants).^28^ The most common location for csPCa was the two posterior quadrants. The accuracy of a thorough mapping biopsy was determined by Crawford who found that TPMB missed only one very small csPCa (1.6%) when compared to whole mount prostatectomy specimens.^29^ Given that the posterior of the prostate is the most common location for PGA a BED of 220 Gy would cause concern that higher radiation doses could expose the anorectum to significant proctitis. However, in a simulation study with prostate phantoms, Vanneste has shown that it is possible to deliver a BED of 220 Gy and protect the rectum with the use of a sculpted rectal spacer to decrease the rectal dose.^27^

While we propose a novel method of determining the nadir PSA after PGA, it is most likely that the ultimate PSA value could go lower. This was noted in the case presented here where the predicted PSA was 2.54 ng/mL while the patient's PSA 3 years after treatment was 0.7 ng/mL. Because brachytherapy delivers some ablative energy outside of the clinical treatment volume, it should be anticipated that the PSA would fall below the calculated value. Thus, the calculated nadir PSA should be considered the upper PSA limit to consider evaluation for recurrence.

While this investigation demonstrated that a biologically effective dose ≥220 Gy ablated 98.5% of the prostate cancer, other energies such as cryotherapy and HIFU may not yield similar results. However, if all csPCa has been identified then other PGA modalities should yield similar results as described in this study. Ultimately, the persistence of csPCa, whether out‐of‐ or infield, should drive the PSA above the nPSA necessitating further evaluation.

## 5 CONCLUSION

This study proposes a novel method to determine the nadir PSA after a successful focal or PGA where the contribution of PSA from the untreated prostate volume can be used to calculate the nadir PSA. Confirmation of this method will require further clinical study. Investigators will need to determine both the volume of prostate that has been treated and the portion that remained untreated. Although this is not standard practice, it should be straightforward to document and to determine the nadir PSA.

## AUTHOR CONTRIBUTIONS

**Nelson N. Stone** and **Vassilios Skouteris**: Project development; data collection; manuscript writing. **Rendi Shu** and **Richard G. Stock**: Manuscript writing. **Ben GL Vanneste**: Project development and manuscript writing. All authors wereinvolved in editing, critical review and final approval of the manuscript.

## CONFLICT OF INTEREST STATEMENT

There are no conflicts of interest for any of the authors.
